# Some Properties of Gold Filling

**Published:** 1893-02

**Authors:** S. H. Guilford


AMERICAN JOURNAL

—OF—

DENTAL SCIENCE.

VOL. XXVI. BALTIMORE, FEBRUARY, 1893. NO. 10.

ARTICLE I.

### SOME PROPERTIES OF GOLD FILLING.

Malleability and ductility are in all cases due to and dependent on cohesion, which may be defined as that force which binds and holds together the ultimate constituents or particles of any solid.

Gold in its pure state possesses this property to such an extent that two masses of it, under suitable conditions, may be as perfectly united in their cold state as they could be if fused by the aid of heat.

If two sheets of pure gold of moderate thickness, with perfectly clean surfaces, be laid one on the other and passed between the rolls of a rolling mill, they will become so thoroughly united that no amount of force can separate them. The same result will take place between a sheet of pure gold and one of pure platina under the same conditions. It is in this way that the crown-metal so largely used to-day in crown- and bridge-work is produced.

So, too, in the process known as fiber-plating, for the production of filaments from which gold lace is woven, a rod of silver is gilded by simply burnishing leaves of pure gold on it. It is then drawn into wire so fine that a length of it extending a mile and a quarter will weigh but one ounce. And how much would the gold on its surface weigh ?

How is this perfect union brought about ? Either one of two theories will account for it. One, the molecular theory, holds that while the attraction of cohesion operates on all solid bodies, its operation is only sensible at insensible distances. When, therefore, the molecules of the same body, or of two similar bodies, are brought within the sphere of this attraction, cohesion takes place.

The other, which for want of a better name we may call the dynamic theory, holds that the molecules of masses of matter are held together by being interlocked with one another, either naturally or by being compelled to assume such a relation under the influence of pressure.

It would seem as though, in the instances cited, both the molecular and dynamic forces operated to produce the result, for union between the metals will not take place unless they are brought into the closest possible apposition, nor will it result if the metals have been hammered or rolled and not subsequently annealed. When a nugget or ingot of gold is subjected to pressure its bulk is sensibly reduced, but when heated to a point slightly below fusion its original dimensions are restored.

This latter process is known as annealing. So, also, when a mass of gold is beaten or rolled it assumes a condition of stiffness and intractability, but its original softness and plasticity are completely restored by annealing. These changes in the mass are explained by the universally conceded fact, that under the influence of pressure the molecules are driven into closer proximity than is natural to them, and that heat, by expanding the mass, allows the particles to move slightly among themselves, and resume, as nearly as may be, their former relations to one another.

Gold, when being beaten into foil, is frequently annealed to restore to it the softness which has been lost in the process of hammering. It also restores its cohesiveness.

Some manufacturers of gold foil have a method of rendering it non-cohesive by a modification of the process. The process of imparting this property to pure gold is kept a secret by the few who understand it. Truly non-cohesive gold possesses the quality of softness or pliability in a remarkable degree. This it could not have without the final annealing after beating, which, in restoring the softness, would also re-establish its cohesiveness. It is therefore reasonable to suppose that it is again deprived of its cohesiveness.

We have seen a cavity filled with non-cohesive gold by pricking it with two cambric needles set in wooden handles. The filling when completed was dense, and the layers of foil were so well united that they could not be separated. While nominally a non-cohesive filling, the layers are really held together by pure cohesion at the point where they are pricked.

Non-cohesive gold has for many years been sold and used under the less distinctive name of soft gold. The latter term, however, is a misnomer, for, as we have stated, all pure gold is soft unless this property has been interfered with by hammering or rolling. No foil can possibly be softer than cohesive foil, but the misuse of the term soft has arisen from the fact that in the manipulation of non-cohesive foil the layers will slide over one another without cohering, which seems to emphasize or exaggerate the impression of softness. The absence of this sliding or gliding quality in cohesive foil naturally but improperly suggests the idea of hardness. In large and accessible cavities, where no necessity exists for the sliding of gold on gold, cohesive foil will be found to be equally as soft and tractable as the non-cohesive variety.—S. H. GUILFORD, in *Cosmos*.

